# Effects of self-monitoring physical activity with a smartphone application combined with physiotherapy coaching in patients with cancer: a randomised controlled trial (SMART-COACH trial)

**DOI:** 10.1007/s11764-025-01862-w

**Published:** 2025-07-15

**Authors:** Harm L. Ormel, Elizabeth H. van Zutphen, Gabriela G. F. van der Schoot, Jourik A. Gietema, Annemiek M. E. Walenkamp

**Affiliations:** Department of Medical Oncology, University Medical Centre Groningen, University of Groningen, Hanzeplein 1, 9713 GZ Groningen, the Netherlands

**Keywords:** Physical activity, Cancer, Healthy lifestyle, Physiotherapy coaching, Smartphone application, EHealth

## Abstract

**Purpose:**

Regardless of the compelling evidence showing that facility-based exercise effectively improves cancer treatment outcome, exercise programs are confronted with low participation and adherence rates. This study aimed to investigate a low-threshold modality to improve self-reported leisure-time physical activity (LTPA).

**Methods:**

In this single-centre, prospective randomised controlled trial, patients living with or beyond cancer were randomised to use the smartphone application “RunKeeper” to self-monitor LTPA with remote physiotherapy coaching for 12 weeks or usual care. The primary endpoint was between-group difference in LTPA at 12 weeks. Secondary endpoints were LTPA measured at 6 and 26 weeks, adhering to the national physical activity guideline, quality of life, cancer-specific functioning and symptoms, self-efficacy, and exercise motivation.

**Results:**

In total, 63 patients with a median age of 46.0 range [34.0 to 57.0] were included. No significant between-group differences were found in LTPA. Significant between-group differences in favour of the group that self-monitored LTPA were found in emotional functioning (*P* = .044), insomnia (*P* = .029) (week 12) and self-efficacy (*P* = .040) (week 26).

**Conclusions:**

Self-monitoring LTPA with RunKeeper and remote physiotherapy coaching did not significantly improve LTPA compared to usual care. The intervention did positively affect self-efficacy, emotional functioning, and insomnia.

**Implications for Cancer Survivors:**

Further research is needed to study the effectiveness of self-monitoring LTPA with remote physiotherapy coaching in a more homogeneous sample of patients with cancer.

**Trial registration:**

(NCT03128333).

**Supplementary Information:**

The online version contains supplementary material available at https://doi.org/10.1007/s11764-025-01862-w.

## Introduction

The importance of lifestyle adaptation during and after cancer treatment is gaining interest [1–3]. Several extensive cohort studies reveal the potential benefits of a healthy lifestyle (e.g. engaging in recommended levels of physical activity (PA), a healthy diet) with an approximately 25% reduction in cancer-specific and overall mortality in patients with breast- and colon cancer compared to an unhealthy lifestyle [4–7]. Also, minimizing cancer treatment-induced long-term effects is crucial to optimise cancer treatment outcomes [8]. This emphasises the need for interventions that focus on secondary prevention such as tailored interventions for long-term lifestyle behaviour change [9–11].

Recent studies have shown that patients are less physically active after cancer diagnosis [12–14]. However, changing lifestyle is not part of standard cancer treatment regimens, and lifestyle adaptation is complex [15–17]. Moreover, patients frequently experience symptoms or limitations of cancer and its treatment that could create barriers to healthy lifestyle behaviour [18, 19]. On the contrary, patients experience a cancer diagnosis as a major life event and evaluation point, which creates an opportunity to change their lifestyle and PA behaviour [15, 20, 21]. There is compelling evidence that shows the beneficial effects of specially supervised facility-based exercise interventions on cancer treatment outcomes [22, 23]. However, well-known disadvantages of facility-based exercise interventions are the time-consuming nature, too demanding participation, high costs, travel distance from the patients’ home to the facility, and low participation rates of patients [24, 25]. Also, the effectiveness and adherence to interventions dilute during the follow-up [26]. A recent study suggests that patients with a lower pre-diagnosis PA level should be offered a low-threshold, low-intensity PA program [17, 27]. Studies show that even small increases in leisure-time physical activity (LTPA) have beneficial health effects [6, 7, 28, 29].

To lower the threshold of a lifestyle intervention, a “smartphone application” (app) as a tool for patients to self-monitor PA can be used in a home-based setting combined with remote physiotherapy coaching by telephone. In the present study, we chose “RunKeeper”, a free, widely spread, and well-known app for self-monitoring PA with over 36 million users. Conveniently, behavioural change techniques to stimulate a healthy lifestyle are integrated well into the RunKeeper app [30], e.g. by self-monitoring PA with GPS or stopwatch, sending daily reminders to get active again, competing with friends, recording progress, goal setting, and personal records.

Therefore, this study aimed to examine whether self-monitoring PA using the RunKeeper app and remote physiotherapy coaching by telephone is superior in improving self-reported LTPA compared to usual care in patients with cancer at 12 weeks after the intervention. Secondary, self-reported LTPA measured at 6 and 26 weeks, adhering to the national PA guideline, health-related quality of life (HRQoL), cancer-specific functioning and symptoms, self-efficacy, and PA motivation at 12 and 26 weeks were examined.

## Methods

### Design and participants

This is a single-centre randomised controlled trial (SMART-COACH trial). Adult patients receiving treatment or in surveillance after their cancer treatment (regardless of type) with a WHO performance score ≤ 1 who were not currently participating in an oncological rehabilitation programme were eligible.

Exclusion criteria were inability to handle or not possess a smartphone (iPhone ≥ 4 or Android phones), inability to read or understand the Dutch language, an active user of the RunKeeper app, and uncontrolled heart- or psychiatric disease.

Written informed consent was obtained from all patients included in the study. The protocol was approved by the local medical ethics committee of the University Medical Centre Groningen (UMCG) and registered on ClinicalTrials.gov (NCT03128333).

### Study procedures and recruitment

Patients were recruited between April 2017 and January 2020 at the Department of Medical Oncology outpatient clinic at the UMCG, the Netherlands. Eligible patients were identified through hospital medical records.

After obtaining written consent, patients visited the hospital for a baseline visit, during which anthropometric measures were taken and their entire PA history was mapped.

After baseline measurements, patients were randomised using computerised randomisation in one of the two study arms, the RunKeeper app, combined with telephone coaching by physiotherapists (intervention group) or usual care (control group). Lifestyle advice was provided as usual care.

Patients underwent study measurements at four time points: baseline (T0), during the intervention at 6 weeks (T1), after the intervention at 12 weeks (T2), and follow-up at 26 weeks (T3). All but one measurement, T0, was completed at home. The T0 measurement was conducted at an outpatient clinic in the UMCG (see Supplementary Fig. 1).

The primary outcome was the difference between groups in self-reported LTPA at T2 compared to T0.

Secondary outcomes were the difference between groups in self-reported LTPA at T3 compared to T0, adherence to the PA guideline as measured by accelerometry, and patient-reported outcomes (PRO), all measured at T2 and T3 compared to T0. Patients were asked to return the accelerometer and patient-reported outcome measures (PROMs) to the principal investigator in pre-stamped envelopes.

### Study intervention

Patients randomised in the intervention group were instructed to use the RunKeeper app for 26 weeks, consisting of 12 weeks of PA intervention, followed by 14 weeks of follow-up. Patients received a brief user manual at T0. They were asked to self-monitor LTPA (e.g. swimming, biking, hiking, running) by GPS or stopwatch function with the RunKeeper app. Moreover, patients were requested to activate the “training reminder” option in the RunKeeper app. In addition, patients were educated about the health risks of a sedentary lifestyle, inactivity, and other unhealthy lifestyle behaviours (e.g. unhealthy diet, overweight or obesity, sun exposure, and alcohol intake). The benefits of a behaviour change, becoming physically active, and pursuing a healthy lifestyle were explained by a trained physiotherapist who coached the patient using behavioural change techniques like motivational interviewing (MI) during the PA program through a biweekly telephone call. The physiotherapist used MI alongside the transtheoretical model (TTM) of behavioural change as a theoretical framework [31]. The MI techniques were tailored to the individual’s stage in the TTM, helping them move towards desired behavioural change by resolving barriers and building self-efficacy. The physiotherapist used a patient-centred approach to optimally tailor the program to patient’s capabilities and preferences. For clarity, patients’ capabilities were not formally assessed but were discussed with the trained physiotherapist during T0 and, together with patients’ preferences, used to set realistic individual goals to optimally tailor the intervention. Detailed information about patients’ LTPA history (for instance, biking, running, tennis) and PA interests were obtained to eliminate barriers that could interfere with becoming physically active and to address and stimulate patients’ internal motivation [32]. Patients were emotionally supported (e.g. encouragements, assurance of capabilities, and successes) and received feedback on their performed LTPA with the RunKeeper app. The physiotherapist closely monitored the patients’ RunKeeper accounts with their email addresses with the patients’ consent. Afterward, each patient was asked to set feasible LTPA goals with the physiotherapist for the upcoming 2 weeks. Plans were evaluated and adjusted if necessary. The physiotherapy coaching telephone calls were tapered off during the 12-week intervention with a 3-month follow-up to examine whether improvements were sustained. The coaching took place in week 1, 3, 7, and 12. We deviated from the tapering protocol when patients could not be reached by telephone.

### Outcome measures

Clinical baseline characteristics were obtained from medical records (e.g. medical history, medication and time after diagnosis). As mentioned previously, patients underwent study measurements at four time points: T0, T1, T2, and T3*.* Self-reported LTPA at T2 compared to T0 was measured by the subscale LTPA sum score of the Physical Activity Scale for the Elderly (PASE) questionnaire. Although originally designed for the elderly, the PASE questionnaire has been validated in adult patients diagnosed with cancer, including younger patients [33]. Scoring components used in the current study are LTPA sum score, total minutes of LTPA (min./wk.), and sedentary time (min./wk.).

The proportion of patients adhering to the Dutch national aerobic PA guideline was determined by using a GT3X-BT accelerometer (ActiGraph, Pensacola, FL, USA) that measured patients’ time spent on moderate- to vigorous physical activity (MVPA) in minutes per week. Patients wore the accelerometer for 1 week at TO. Data was collected with a sampling rate of 60 Hz. The accelerometer registered raw acceleration data gravity units, Euclidean norm minus 1 (ENMO) [34]. The Actilife Software version 6.13.4 was used to download and convert raw data into CSV files. CSV files were processed using the GGIR package version 1.6 in R’ [34]. Cut-points used for determining MVPA were: ≥ 69 mG (hip-worn), ≥ 100 mG (wrist-worn, non-dominant side) and ≥ 110 mG (wrist-worn, dominant side) [35, 36]. A valid day of wearing time was defined as a minimum of 8 h [37].

Diastolic and systolic blood pressure, skinfold measurements, along with other anthropometric measures, were assessed only at baseline (T0). Diastolic and systolic blood pressure were measured in rest after patients sat quietly for > 5 min. BMI (kg/m^2^) was calculated using weight and height. A Harpenden Skinfold Calliper measured fat percentage as determined by a 4-site skinfold measurement [38].

Patient-reported HRQoL, as well as cancer-related functioning and symptoms, were measured with the European Organization for Research and Treatment of Cancer (EORTC) Quality of Life Questionnaire Core 30 (QLQ-C30) [39]. Scores for each scale range from 0 to 100. For functional and global health status scales, higher scores indicate better functioning or quality of life, whereas for symptom scales, higher scores indicate more severe symptoms.

Patient-reported self-efficacy was measured by the “Algemene Competentie Schaal” (ALCOS), the validated Dutch version of the General Self-Efficacy Scale (GSES) [40]. Responses were presented on a 5-point Likert scale (totally disagree, disagree, not disagree/not agree, agree and totally agree). The points per item range from 1 to 5, with total score range from 16 to 90, where a higher score represents a higher level of self-efficacy.

Exercise motivation was measured by categorising patients as non-regular exercisers (pre-contemplation, contemplation, preparation, relapse) or as regular exercisers (action, maintenance) using the TTM stage of behaviour change [31].

### Statistical analyses

The power analysis was based on a pilot study investigating the effect of the RunKeeper app use on LTPA compared to usual care in a 12-week program, providing the estimated standardized effect size (*d* = 0.715) [41]. Enrolment of 31 patients per group (62 in total) provided 80% power with a one-sided significance *P* = 0.05 using the estimated standardized effect size (*d* = 0.715) to detect an improvement in PASE LTPA Sum Score at T2 of 36.8, anticipating a 15% dropout. The maximum score of the LTPA sum score is 502.

Intention-to-treat linear mixed model analyses were used to model the continuous outcomes at T1, T2, and T3, assuming an unstructured correlation structure, and to compare the average mean between-group differences and were checked for normality using a PP-plot. Our analyses were adjusted for baseline values, age, and treatment purpose because of disparities in these covariates and their possible associations with outcomes. In this study, curative treatment was defined as treatment with the intent to eliminate the cancer. Palliative treatment was defined as care aimed at improving the quality of life of patients and their families facing life-threatening illness, through the prevention and relief of suffering by means of early identification and impeccable assessment and treatment of pain and other physical, psychosocial, and spiritual problems in line with the Dutch national framework for palliative care (modified WHO-2002 definition). Exploratory post hoc subgroup analyses were performed of within- and between-group differences in LTPA sum score per treatment purpose. Standardized effect size (ES) was considered to be trivial, low, moderate, and high and described by Cohen’s *D* as lower than 0.2, 0.2–0.5, 0.5–0.8, or greater than 0.8 [42].

All data over time points T0, T1, T2, and T3 were analysed using the Statistical Package of Social Sciences (SPSS, version 22.0) and R (R Core Team 2019, Language and Environment for Statistical Computing, version 3.6.2, Vienna, Austria, Europe).

## Results

### Study population

**Fig. 1:**
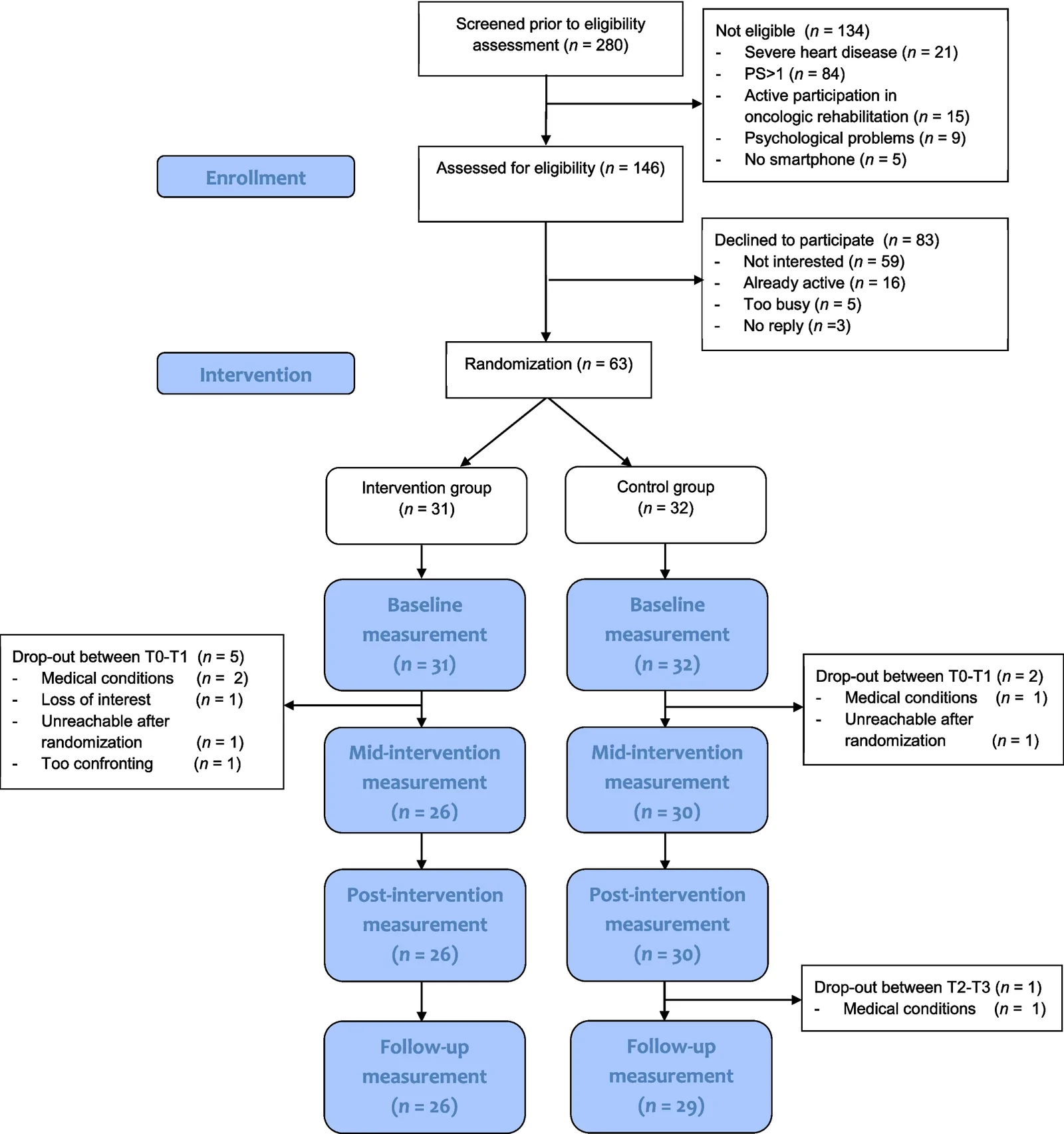
CONSORT diagram. PS, performance score; BMI, body mass index

Patient characteristics are shown in Table 1. In total, 63 patients were enrolled. Patients were seen for baseline assessment at a median of 63.0 months [31.0 to 107.0] and 27.0 months [9.5 to 63.0] after diagnosis in the intervention- and control groups, respectively (*P* = 0.021). Figure 1 displays the CONSORT flow diagram. The dropout rate was 14.9% from T0 to T1 and 16.3% from T0 to T2. No intervention-related adverse events were reported.

**Table 1:** Baseline characteristics of patients participating in the SMART-COACH trial (*n* = 63)

Characteristics mean (SD (±)), median [IQR], no. (%)	Intervention group (*n* = 31)	Control group (*n* = 32)
*Demographic characteristics*		
Age at inclusion (years)	41.9 ± 12.8	49.3 ± 13.4
Marital status		
Married/relationship (living together)	21 (67.7)	24 (75.1)
Single/relationship (not living together)	10 (32.3)	8 (25.0)
Gender (male)	24 (77.4)	22 (68.8)
Education		
Low-medium (elementary-, primary- or secondary school/lower- or secondary vocational education)	18 (58.1)	21 (65.6)
High (higher vocational-, college- or university education)	13 (41.9)	11 (34.4)
Employment status		
Paid work/work as a volunteer/student	27 (87.1)	24 (75.1)
Sickness leave/re-integration process	1 (3.2)	3 (9.4)
Unemployed/retirement/housewife	3 (9.7)	5 (15.6)
*Behavioural characteristics*		
Current or former smoker (yes)	12 (38.7)	20 (62.5)
Alcohol consumption (yes)	21 (67.7)	17 (53.1)
*Anthropometrics*		
Weight (kg)	90.6 ± 14.2	88.7 ± 10.3
BMI (kg/m^2^)	27.9 ± 4.2	28.1 ± 5.8
Waist circumference (cm)	95.7 ± 11.9	97.0 ± 15.1
Hip circumference (cm)	105.2 ± 13.7	105.0 ± 12.6
Fat percentage (%)	29.3 ± 9.5	30.8 ± 9.3
Systolic blood pressure (mmHg)	128.3 ± 11.5	129.9 ± 8.8
Diastolic blood pressure (mmHg)	78.9 ± 8.8	79.2 ± 10.1
*Medical characteristics*		
Cancer type		
Testicular cancer	18 (58.1)	12 (37.5)
Breast cancer	5 (16.1)	4 (12.5)
Neuroendocrine tumour	3 (9.7)	7 (21.9)
Glioma	2 (6.3)	2 (6.5)
Other	3 (9.6)	7 (21.8)
Cancer stage		
I	3 (9.7)	5 (15.6)
II	15 (48.4)	12 (43.8)
III	3 (9.7)	1 (3.1)
IV	11 (35.4)	11 (34.4)
Treatment purpose		
Curative	21 (67.7)	18 (56.3)
Adjuvant	2 (6.5)	4 (12.5)
Palliative	8 (25.8)	10 (31.3)
Radiation therapy (yes)	7 (22.6)	7 (21.9)
Chemotherapy (yes)	27 (87.1)	20 (62.5)
Time since diagnosis (months)	63.0 [31.0 to 107.0]	27.0 [9.5 to 63.0]

*SD*, standard deviation; *IQR*, interquartile range; *BMI*, body mass index

### Leisure-time physical activity

**Fig. 2:**
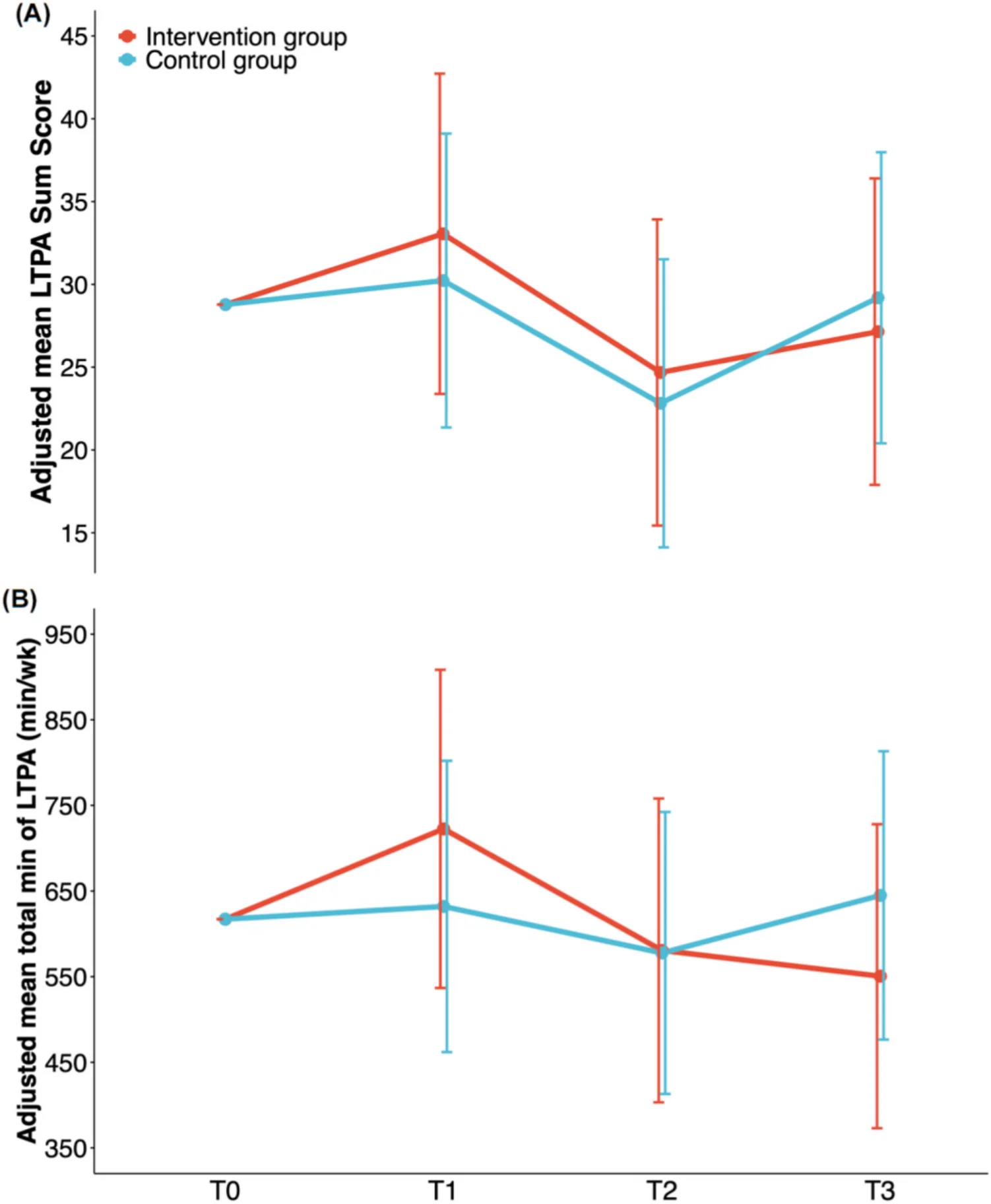
Change in self-reported LTPA as measured by the PASE questionnaire. **A** Adjusted mean difference in PASE subscale LTPA sum score. **B** Adjusted mean difference in PASE subscale Total minutes of LTPA. PASE, physical activity scale for the elderly; LTPA, leisure-time physical activity; min, minutes; wk, week; T0, baseline; T1, during intervention (6 weeks); T2, postintervention (12 weeks); T3, follow-up (26 weeks)

The LTPA sum score and LTPA minutes per week are depicted in Fig. 2 and Supplementary Table 1. At T0, the LTPA sum score was scored 25.4 (standard error (SE) 5.3) and 24.8 (SE 5.4) by the intervention group and control group, respectively. An improvement from T0 to T1 was found in the LTPA sum score in both groups with a non-significant between-group adjusted mean difference (AMD) of 2.8 (ES 0.12, *P* = 0.330), a trivial positive effect. This change was not maintained at T2 in both groups. At T3, an increase in LTPA sum score was found in both groups compared to T0 with a non-significant between-group AMD of − 2.1 (ES − 0.09, *P* = 0.370). The exploratory analyses of LTPA sum score per treatment purpose groups (curative, adjuvant and palliative) are depicted in Supplementary Table 1 and Supplementary Fig. 2.

### Secondary outcomes

#### Adherence to the national PA guideline

**Fig. 3:**
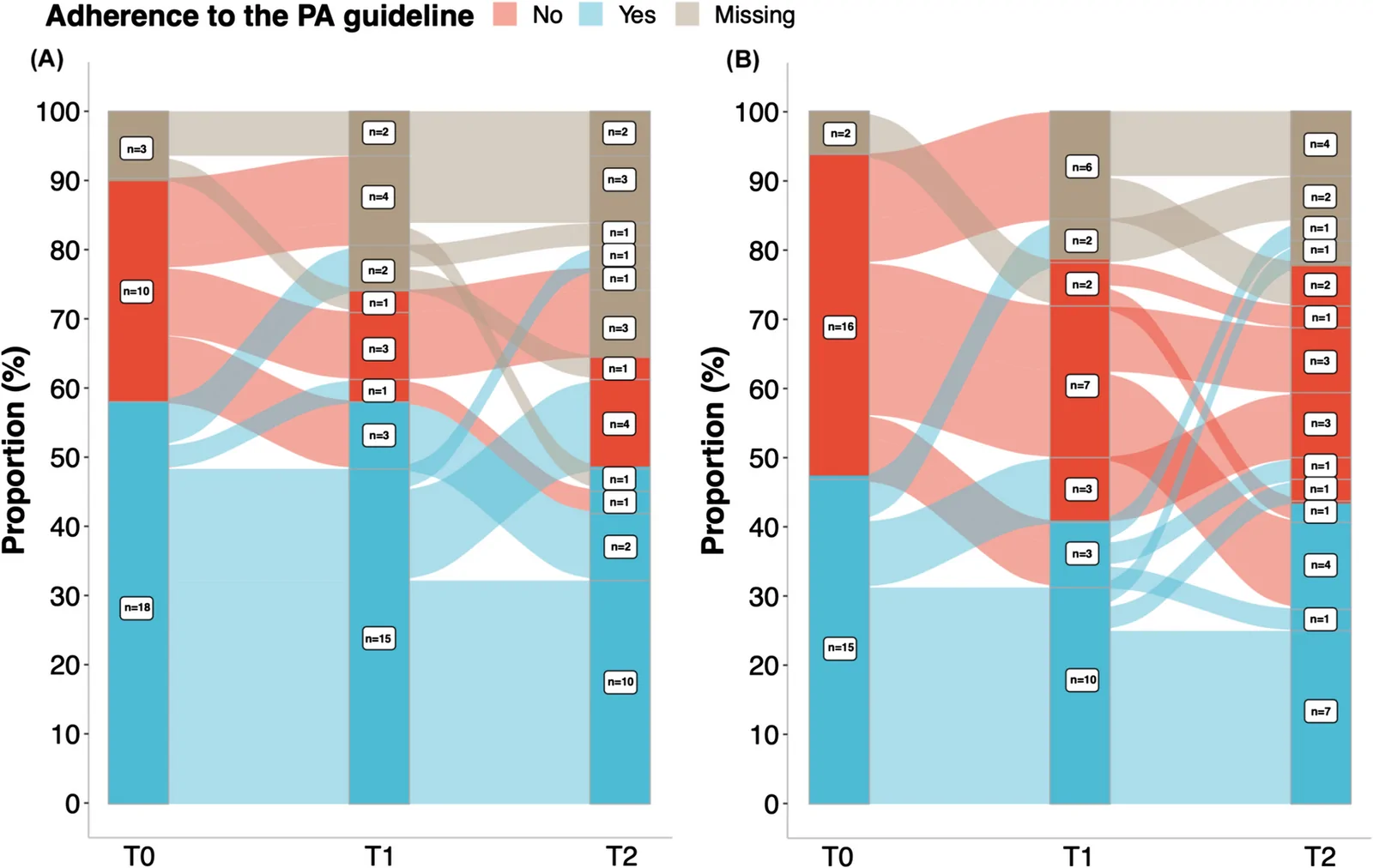
Change in adherence to the PA guideline. **A**, Intervention group; **B**, Control group. Alluvial diagram of proportion of patients adhering to the national PA guideline. Flows between the blocks represent changes in clusters of patients that adhere or do not adhere to the PA guideline over time. Missing data reflect missing measurements due to patient dropouts or measurement errors, as analyses were conducted on an intention-to-treat basis. PA, physical activity; T0, baseline; T2, postintervention (12 weeks); T3, follow-up (26 weeks)

At T0, the proportion of patients that were adhering to the PA guideline was 58.1% (*n* = 18) in the intervention group and 46.9% (*n* = 15) in the control group (see Fig. 3). At T2, adherence remained stable in the intervention group and decreased in the control group, 40.6% (*n* = 13). From T2 to T3, adherence decreased in the intervention group to 48.4% (*n* = 13) and remained the same in the control group, 40.6% (*n* = 13). There were no significant between-group differences in proportional changes.

#### HRQoL and cancer-related functioning and symptoms

At T0, mean HRQoL was rated 76.4 (SE 3.0) in the intervention group and 76.1 (SE 2.9) in the control group. No between-group differences were found at T2 or T3 in HRQoL. From T0 to T2, HRQoL increased significantly in the intervention group compared to a non-significant increase in the control group with within-group differences of 4.6 (95% confidence interval (CI) 0.0 to 9.2) and 1.1 (95%CI −3.1 to 5.2), respectively. At T3, HRQoL increased significantly in the control group in contrast to a non-significant increase in the intervention group with within-group differences of 6.6 (95%CI 1.0 to 12.3) and 3.6 (95%CI − 2.4 to 9.5). At T2, significant between-group differences were found in favour of the intervention group in emotional functioning (*P* = 0.044) and insomnia (*P* = 0.029). Changes in HRQoL and cancer-specific functioning and symptoms are depicted in Supplementary Table 2.

#### Self-efficacy

At T0, the ALCOS questionnaire’s mean sum score was 64.6 (SE 3.0) in the intervention group and 76.1 (SE 2.9) in the control group. From T0 to T2, self-efficacy increased in the intervention group compared to a decrease in the control group with a between-group AMD of 2.0 (ES 0.18, *P* = 0.171). A significant between-group AMD of 3.8 (ES 0.34, *P* = 0.040) was found at T3 in favour of the intervention group, with an increase in self-efficacy in the intervention group compared to a decrease in self-efficacy in the control group. Changes in self-efficacy are depicted in Supplementary Table 3).

#### Exercise motivation

At T0, the proportion of patients that were regular exercisers and motivated to engage in LTPA was 30% (*n* = 9) in the intervention group and 32.3% (*n* = 10) in the control group. At T2, the proportion of regular exercisers increased in the intervention group to 40.0% (*n* = 12) and improved in the control group to 45.2% (*n* = 14). From T2 to T3, the proportion of regular exercisers decreased in the intervention group to 33.3% (*n* = 10) and the control group to 32.3% (*n* = 10). There were no significant between-group differences in proportional changes.

## Discussion

In the SMART-COACH trial, patients diagnosed with cancer who self-monitored PA using the RunKeeper app and physiotherapy coaching did not improve self-reported LTPA compared to usual care directly after the intervention at 12 weeks. The study showed beneficial effects on patient-reported emotional functioning and insomnia directly after the intervention at 12 weeks and on patient-reported self-efficacy at 26-week follow-up compared to the control group.

Low-threshold interventions like those investigated in this study are urgently needed, as supervised facility-based exercise interventions are difficult to access due to high travel burdens, time constraints, and a growing group of patients cannot afford such a program due to limited coverage of costs by health insurances [43–45]. Our low-threshold intervention enabled patients with cancer to perform LTPA in a supervised home-based setting.

We did not find an increase in LTPA after 6 weeks in the intervention group compared to the control group in contrast to the significant increase in the feasibility study [41]. The difference in effect might have been caused by differences in the characteristics of the study population. In a study investigating 110 patients with a mild-to-moderate mobility disability, the effect on MPVA was evaluated of an app-based intervention consisting of a 12-week walking and exercise program and three face-to-face consultations compared with a supervised exercise and health coaching program [46]. The app-based program aimed at engaging patients in at least 30 min of MVPA per day. No difference in effectiveness between groups was observed. The authors concluded that both interventions did not increase MVPA but that app-based interventions could be an alternative for supervised exercise programs to improve health-related outcomes [46]. Another study evaluated the effect of a 12-week home-based exercise program consisting of prescribed aerobic and resistance exercises with an additional smartphone app coupled with a pedometer compared to a regular home-based exercise program where patients receive the same prescriptions and a brochure in patients with breast cancer after completion of primary cancer treatment [47]. Both interventions effectively improved physical function, PA level, and QoL. However, there were no significant between-group differences. Similar to our intervention, they used a smartphone app for 12 weeks.

A recent single-arm study in 269 breast cancer survivors found a significant increase in MVPA after the intervention at 12 weeks compared to baseline as a result of a custom-designed Fit2Thrive self-monitoring PA app, Fitbit, and additional components (e.g. telephone support calls, online gym and text messages/push notifications) [48]. The use of the Fit2Thrive app to self-monitor PA seems promising. Our intervention was similarly designed based on behaviour change theory, particularly the transtheoretical model stage of behaviour change and social cognitive theory. In our intervention, we enhanced patients’ awareness of their LTPA behaviour and to supported progression through the stages of change by increasing self-efficacy.

We observed a small effect of the intervention on patient-reported self-efficacy. The coaching by a trained physiotherapist or self-monitoring LTPA with RunKeeper could have contributed to the increase in self-efficacy. Patients were repeatedly questioned and triggered during the coaching sessions on how they could become more physically active. Self-reflection on their LTPA behaviour, and setting new goals could have contributed to a gain in self-awareness, self-confidence, motivation, and self-efficacy. A higher sense of self-efficacy might indicate that patients were more confident due to the self-monitoring of LTPA using the RunKeeper app and physiotherapy coaching remotely. Findings of a recently published study of Westerhuis et al. support our arguments that such blended interventions can enhance patients’ motivation, self-awareness, and confidence in managing their physical activity behaviour, thereby improving self-efficacy [49]. In addition, our intervention had a significant positive effect on self-reported emotional functioning in comparison with the control group as measured directly after the intervention. A life-threatening cancer diagnosis can strongly affect the emotional functioning of patients, families, and caregivers [50]. Guidance in the form of coaching by a trained physiotherapist as well as being able to actively contribute to their own physical health for patients by self-monitoring LTPA, could both have positively affected patients’ emotional functioning in the present study. Findings in comparable studies support the beneficial effect of exercise interventions on emotional functioning in cancer patients [51, 52]. We found moderate effect sizes for self-reported emotional functioning and insomnia (sleep disturbance) in favour of the intervention group. The positive effect of our intervention on insomnia is in line with the results of a meta-analysis of nine randomised controlled trials that investigated moderate-intensity walking exercise in patients with cancer [53].

Strengths of our study include the randomised controlled design, innovative approach using modern technology, and physiotherapy coaching. To enhance implementation, we used an existing, widespread, free smartphone app to self-monitor LTPA.

Limitations of this study are the inclusion of patients with multiple cancer types, treatment purposes and regimens that could have altered the effects of the intervention. The variable time after diagnosis varied greatly among patients that were included with a significant difference between groups due to low patient numbers. A significant difference in the variable time after diagnosis was measured between groups at T0. This may have influenced patients’ readiness or ability to engage in LTPA and should be considered when interpreting the results. Another limitation is the use of the PASE to measure the primary outcome LTPA instead of objective accelerometry. Patient-reported outcome measures could be prone to biases like recall bias and social desirability bias [54]. Additionally, the short intervals between repeated measurements (T0–T1 and T1–T2) may have introduced response fatigue or sensitisation effects, potentially influencing the reliability of self-reported data. Future studies could consider longer intervals or incorporate objective measures to mitigate these risks. Lastly, contamination in the control group through spontaneous adoption of LTPA cannot be ruled out.

The advantage of a supervised remote home-based intervention rather than a facility-based supervised exercise intervention lies in the capability of healthcare professionals to monitor and coach patients’ health behaviour in their free living environment. Also, there could be several other advantages such as no travel burden for patients, less time consuming, no patient visits at an outpatient clinic and low costs. During the COVID-19 pandemic, physical distancing and hygiene were especially important to take into account for vulnerable cancer patients. Lock-downs all over the globe limited participation in facility-based supervised exercise interventions [55]. This emphasizes the need for eHealth interventions like the one we have investigated in the SMART-COACH trial [56].

## Conclusions

Self-monitoring PA with the RunKeeper app with telephone coaching by physiotherapists did not result in a significant improvement in LTPA compared to usual care. The intervention did have a positive effect on patient-reported self-efficacy, emotional functioning, and insomnia.

## Supplementary Information

Below is the link to the electronic supplementary material.

Supplementary file1 (DOCX 555 KB)

## Data Availability

All datasets used and analysed during the current study are available from the corresponding author on reasonable request.
