# Supplementary material for: Effects of self-monitoring physical activity with a smartphone application combined with physiotherapy coaching in patients with cancer: a randomised controlled trial (SMART-COACH trial)
Source: J Cancer Surviv. 2025 Jul 15;20(Suppl 1):799–809. doi: 10.1007/s11764-025-01862-w (PMC13676569; doi:10.1007/s11764-025-01862-w)
Supplement: Supplementary file 1 [file 11764_2025_1862_MOESM1_ESM.docx]

**Supplementary Material**

*

*

*

*

*

*

*

**(B)**

**(A)**

**Supplementary figure 1.** Design of the SMART-COACH trial.

Abbreviations: PASE, physical activity scale for the elderly; LTPA, leisure-time physical activity; PA, physical activity; EORTC, European Organization for Research and Treatment of Cancer; QLQ-C30; Quality of Life Questionnaire Core 30; ALCOS, general self-efficacy scale; T0, baseline; T1, during-intervention (6 weeks); T2, post-intervention (12 weeks); T3, follow-up (26 weeks).

**Supplementary Table 1**. Results of LTPA sum score per treatment purpose.

|  | | Within-group differences from T0 | | |  | Between-group differences from T0 | | |
| --- | --- | --- | --- | --- | --- | --- | --- | --- |
| PASE LTPA | T0 | Δ T0-T1* | Δ T0-T2* | Δ T0-T3* |  | Δ T0-T1** | Δ T0-T2** | Δ T0-T3** |
| Sum Score | Mean (SE) | Mean (95% CI) | Mean (95% CI) | Mean (95% CI) |  | Mean; P; ES | Mean; P; ES | Mean; P; ES |
| Curative | | | | | | | | |
| IG (*n* = 21) CG (*n* = 18) | 29.0 (6.6)  30.2 (7.0) | **15.5 (1.5 to 29.5)**  9.2 (-3.7 to 22.2) | 2.0 (-13.7 to 17.7) 1.1 (-15.0 to 17.1) | 5.6 (-11.6 to 22.8) 2.9 (-14.7 to 20.5) | | 9.6; .148; 0.35 | 4.2; .308; 0.15 | 6.3; .226; 0.23 |
| Adjuvant | | | | | | | | |
| IG (*n* = 2) CG (*n* = 4) | 36.1 (11.4)  21.3 (9.8) | -14.8 (-42.7 to 13.0) 13.8 (-10.3 to 37.9) | -22.8 (-53.7 to 8.2) 5.3 (-21.5 to 32.0) | -15.7 (-47.7 to 16.3) 16.0 (-11.8 to 43.7) | | -9.4; .238; -0.32 | -8.8; .253; -0.30 | -12.4; .176; -0.42 |
| Palliative | | | | | | | | |
| IG (*n* = 8) CG (*n* = 10) | 28.8 (7.9)  28.3 (7.1) | 0.4 (-16.9 to 17.7) 2.4 (-12.6 to 17.3) | 2.2 (-19.8 to 24.1) -3.3 (-20.8 to 14.2) | -1.2 (-25.0 to 22.5) 11.1 (-8.4 to 30.6) | | -2.3; .447; -0.10 | 3.2; .373; 0.14 | -12.4; .107; -0.54 |

Abbreviations: LTPA, leisure-time physical activity; T0 baseline; T1, during-intervention; T2, post-intervention; T3, follow-up (26 weeks); IG, intervention group; CG, control group; SE, standard error; CI, confidence interval; ES, effect size.
*Mixed models were used to assess within-group effects from baseline to T1, T2 and T3, adjusted for age.
**Mixed models were also used to assess between-group effects from baseline to T1, T2 and T3, adjusted for baseline values: LTPA Sum Score: 27.7 in the curative group, 27.6 in adjuvant group, 31.8 in the palliative group as well as for age: 39.5 in the curative group, 53.9 in the adjuvant group and 56.4 in the palliative group.

**Supplementary table 2**. Results of HRQoL and cancer-specific functioning and symptoms as measured by the EORTC QLQ-C30 questionnaire.

|  |  | Within-group differences from T0 | |  | Between-group differences from T0 | |
| --- | --- | --- | --- | --- | --- | --- |
| EORTC QLQ | T0 | Δ T0-T2* | Δ T0-T3* |  | Δ T0-T2** | Δ T0-T3** |
| scales | Mean (SE) | Mean (95% CI) | Mean (95% CI) |  | Mean; P; ES | Mean; P; ES |
| HRQoL | |  |  |  |  |  |
| IG  CG | 76.4 (3.0)  76.1 (2.9) | **4.6 (0.03 to 9.2)**  1.1 (-3.1 to 5.2) | 3.6 (-2.4 to 9.5)  **6.6 (1.0 to 12.3)** | | 3.9; .109; 0.22 | -3.0; .181; -0.17 |
| Physical functioning | | |  |  |  |  |
| IG  CG | 90.0 (2.3)  88.4 (2.3) | 0.9 (-2.9 to 4.7)  2.2 (-1.3 to 5.6) | 1.3 (-3.6 to 6.1)  **4.8 (0.2 to 9.4)** | | -0.7; .369; -0.05 | -3.0; .090; -0.22 |
| Role functioning | | |  |  |  |  |
| IG  CG | 78.9 (4.0)  79.6 (3.9) | **11.4 (3.8 to 18.9)**  6.7 (-0.2 to 13.6) | 8.7 (-0.8 to 18.1)  **10.1 (1.1 to 19.1)** | | 4.6; .137; 0.19 | -2.6; .277; -0.11 |
| Emotional functioning | | |  |  |  |  |
| IG  CG | 85.6 (3.3)  83.9 (3.3) | 4.1 (-3.3 to 11.4)  -3.6 (-10.3 to 3.2) | 4.0 (-4.8 to 12.7)  1.7 (-6.7 to 10.0) | | **8.3; .022; 0.43** | 2.0; .321; 0.10 |
| Cognitive functioning | | |  |  |  |  |
| IG  CG | 87.8 (4.1)  79.0 (4.1) | 2.1 (-6.4 to 10.6)  1.3 (-6.5 to 9.0) | 2.3 (-8.1 to 12.6)  2.2 (-7.7 to 12.1) | | 4.1; .195; 0.17 | 3.4; .245; 0.14 |
| Social functioning | | |  |  |  |  |
| IG  CG | 82.8 (4.4)  82.3 (4.3) | 4.4 (-2.5 to 11.2)  2.5 (-3.7 to 8.7) | 5.4 (-3.5 to 14.3)  5.4 (-3.0 to 13.8) | | 3.1; .215; 0.12 | 0.2; .477; 0.01 |
| Fatigue | | |  |  |  |  |
| IG  CG | 30.7 (4.4)  33.0 (4.3) | -6.0 (-12.5 to 0.6)  -6.1 (-12.0 to 0.2) | -4.2 (-12.8 to 4.3)  **-10.8 (-18.9 to -2.8)** | | -1.4; .375; -0.05 | 5.1; .125; 0.19 |
| Nausea and vomiting | | |  |  |  |  |
| IG  CG | 2.2 (1.5)  4.3 (1.4) | -0.1 (-4.5 to 4.3)  -3.2 (-7.3 to 0.9) | -1.5 (-5.9 to 2.8)  -1.8 (-6.0 to 2.3) | | 1.0; .264; 0.09 | -1.8; .136; -0.17 |
| Pain | | |  |  |  |  |
| IG  CG | 11.1 (3.1)  8.6 (3.1) | -2.1 (-8.4 to 3.1)  0.2 (-5.6 to 5.9) | -4.9 (-12.7 to 2.8)  -3.7 (-11.0 to 3.7) | | -1.2; .376; -0.06 | -0.01; .500; 0.00 |
| Dyspnea | | |  |  |  |  |
| IG  CG | 10.0 (2.9)  10.8 (2.9) | -1.7 (-7.4 to 3.9)  -3.6 (-8.8 to 1.5) | -2.2 (-9.2 to 4.9)  -2.3 (-9.0 to 4.4) | | 1.4; .352; 0.08 | -0.4; .458; -0.02 |
| Insomnia | | |  |  |  |  |
| IG  CG | 13.3 (4.4)  15.1 (4.3) | -3.1 (-11.9 to 5.5)  **8.8 (0.9 to 16.8)** | 6.2 (-4.5 to 17.0)  2.4 (-7.7 to 12.7) | | **-12.5; .015; -0.52** | 2.9; .311; 0.12 |
| Appetite loss | | |  |  |  |  |
| IG  CG | 4.4 (2.6)  5.4 (2.6) | 1.2 (-4.6 to 7.1)  0.002 (-5.4 to 5.4) | -1.7 (-8.6 to 5.3)  -0.2 (-6.9 to 6.4) | | 0.8; .409; 0.05 | -2.2; .275; -0.15 |
| Constipation | | |  |  |  |  |
| IG  CG | 6.7 (2.9)  4.3 (2.8) | -1.1 (-5.7 to 3.5)  -0.3 (-4.5 to 4.0) | 0.9 (-5.1 to 6.8)  2.1 (-3.5 to 7.8) | | 0.2; .474; 0.01 | -0.2; .479; -0.01 |
| Diarrhea | | |  |  |  |  |
| IG  CG | 5.6 (2.8)  5.4 (2.8) | 1.9 (-4.5 to 8.4)  2.3 (-3.6 to 8.3) | 0.2 (-7.4 to 7.8)  2.2 (-5.1 to 9.4) | | -0.2; .481; -0.01 | -2.0; .314; -0.13 |
| Financial difficulties | | |  |  |  |  |
| IG  CG | 8.9 (3.5)  8.6 (3.5) | 0.3 (-6.3 to 6.9)  0.1 (-6.0 to 6.2) | -2.7 (-11.0 to 5.6)  -1.9 (-9.8 to 6.0) | | -0.2; .480; -0.01 | -0.1; .488; -0.01 |

Abbreviations: EORTC, European Organization for Research and Treatment of Cancer; QLQ-C30, Quality of Life Questionnaire Core 30; HRQoL, health-related quality of life; T0, baseline; T2, post-intervention (12 weeks); T3, follow-up (26 weeks); IG, intervention group; CG, control group; SE: standard error.*Mixed models were used to assess within-group effects from baseline to T2 and T3.
**Mixed models were also used to assess between-group effects from baseline to T2 and T3, adjusted for baseline values: HRQoL: 78.7, Physical functioning: 90.6, Role functioning: 80.3, Emotional functioning: 86.9, Cognitive functioning: 84.3, Social functioning: 85.4, Fatigue: 29.2, Nausea and vomiting: 2.9, Pain: 8.6, Dyspnea: 9.8, Insomnia: 11.4, Appetite loss: 3.8, Constipation: 4.4, Diarrhea: 4.4, Financial difficulties: 7.6. For the functional scales a positive value indicates an improvement that favors the IG, while for the symptom scales it is the opposite as a negative value indicates an improvement.

**Supplementary table 3**. Results of self-efficacy as measured by the ALCOS questionnaire.

|  |  | Within-group differences from T0 | |  | Between-group differences from T0 | |
| --- | --- | --- | --- | --- | --- | --- |
| ALCOS | T0 | Δ T0-T2* | Δ T0-T3* |  | Δ T0-T2** | Δ T0-T3** |
| scales | Mean (SE) | Mean (95% CI) | Mean (95% CI) |  | Mean; P; ES | Mean; P; ES |
| Sum score | |  |  |  |  |  |
| IG  CG | 64.6 (2.0)  64.2 (2.0) | 1.6 (-2.0 to 5.2)  -0.5 (-3.7 to 2.7) | 2.1 (-2.5 to 6.6)  -1.4 (-5.7 to 2.9) | | 2.0; .171; 0.18 | **3.8; .040; 0.34** |
| Competence | | |  |  |  |  |
| IG  CG | 23.9 (0.9)  24.1 (0.8) | 0.6 (-0.8 to 2.1)  0.1 (-1.1 to 1.4) | 1.2 (-0.6 to 3.0)  -0.1 (-1.8 to 1.6) | | 0.5; .292; 0.11 | 1.3; .070; 0.28 |
| Initiative | | |  |  |  |  |
| IG  CG | 15.4 (0.7)  15.4 (0.7) | 0.1 (-1.3 to 1.4)  -0.6 (-1.8 to 0.7) | 0.6 (-1.1 to 2.2)  -0.9 (-2.5 to 0.7) | | 0.6; .207; 0.15 | **1.7; .017; 0.43** |
| Persistence | | |  |  |  |  |
| IG  CG | 24.8 (0.7)  25.1 (0.7) | 0.8 (-0.6 to 2.2)  -0.02 (-1.3 to 1.2) | 0.3 (-1.4 to 2.0)  -0.4 (-2.0 to 1.2) | | 0.8; .183; 0.20 | 0.7; .210; 0.17 |

Abbreviations: T0, baseline; T2, post-intervention (12 weeks); T3, follow-up (26 weeks); IG, intervention group; CG, control group; CI, confidence interval SE: standard error.
*Mixed models were used to assess within-group effects from baseline to T2 and T3.
**Mixed models were also used to assess between-group effects from baseline to T2 and T3, adjusted for baseline values: Sum score: 64.4, Competence: 24.1, Initiative: 15.4, Persistence: 25.0.

**Supplementary figure 2.** Change in self-reported LTPA measured by the PASE questionnaire in the SMART-COACH trial per treatment purpose. (A) Curative; (B) Adjuvant (C) Palliative.

Abbreviations: PASE, physical activity scale for the elderly; LTPA, leisure-time physical activity; T0, baseline; T1, during-intervention (6 weeks); T2, post-intervention (12 weeks); T3, follow-up (26 weeks).
